# Arterial stiffening acts synergistically with APOE genotype and AD biomarker status to influence memory in older adults without dementia

**DOI:** 10.1186/s13195-021-00851-2

**Published:** 2021-07-01

**Authors:** Katherine J. Bangen, Denis S. Smirnov, Lisa Delano-Wood, Christina E. Wierenga, Mark W. Bondi, David P. Salmon, Douglas Galasko

**Affiliations:** 1 Research Service, VA San Diego Healthcare System, Building 13, 3350 La Jolla Village Drive (151A), San Diego, CA 92161 USA; 2 Department of Psychiatry, University of California, San Diego, La Jolla, CA USA; 3 Medical Scientist Training Program, University of California, San Diego, La Jolla, CA USA; 4 Department of Neurosciences, University of California, San Diego, La Jolla, CA USA; 5 Psychology Service, VA San Diego Healthcare System, San Diego, CA USA

**Keywords:** Alzheimer’s disease, Pulse wave velocity, Arterial stiffening, Vascular risk factors, Cognition, Memory, Microcirculation, Aging

## Abstract

**Background:**

Arterial stiffening has emerged as an important risk factor for Alzheimer’s disease (AD) and related dementias. Carotid-femoral pulse wave velocity has been proposed as a non-invasive and reproducible method to assess arterial stiffness. However, the association of pulse wave velocity with performance across multiple cognitive domains as well as interactions with in vivo AD biomarkers and apolipoprotein E (APOE) genotype has received limited study.

**Method:**

We studied 193 older adult volunteers (167 with normal cognition and 26 with mild cognitive impairment) who underwent comprehensive medical and neuropsychological evaluation at the University of California, San Diego Alzheimer’s Disease Research Center. Cerebrospinal fluid (CSF) biomarkers were available on 123 participants (63%). Linear models examined whether pulse wave velocity significantly interacted with APOE ε4 status and CSF AD biomarker positivity (based on the ratio of total tau over beta-amyloid [tau/Aβ_42_]) on memory, language, executive functioning, attention, and visuospatial abilities.

**Results:**

After adjusting for demographic characteristics and vascular risk burden, across the entire sample, pulse wave velocity was associated with poorer executive functioning but not the performance in the other cognitive domains. When the modifying effects of AD genetic risk and CSF AD biomarkers were considered, pulse wave velocity interacted with APOE genotype and CSF tau/Aβ ratio such that a stronger association between elevated pulse wave velocity and poorer memory performance was found among those positive for CSF and genetic AD markers. There were no significant interaction effects for non-memory cognitive domains.

**Conclusion:**

The findings suggest that pulse wave velocity, a non-invasive method to assess arterial wall properties, may be a useful marker of risk for cognitive decline, particularly among individuals who are APOE ε4 carriers or CSF AD biomarke0r-positive.

**Supplementary Information:**

The online version contains supplementary material available at 10.1186/s13195-021-00851-2.

## Introduction

Risk factors for cerebrovascular disease can also increase the risk of developing Alzheimer’s disease (AD) and are potentially modifiable to prevent or delay cognitive impairment [1, 2]. Arterial stiffness underlies the effect of blood pressure on the brain and has emerged as an important non-traditional vascular risk factor [3, 4]. Arterial stiffening occurs with aging and is exacerbated by conditions such as hypertension and diabetes [5]. Pulse wave velocity (PWV) is a non-invasive and reproducible method to assess arterial stiffness [6] that has been linked to increased cerebral small vessel disease evidenced by white matter hyperintensities on magnetic resonance imaging (MRI) and to β-amyloid (Aβ) accumulation associated with AD [1]. Indeed, increased PWV (i.e., greater arterial stiffness) has been proposed as a possible mechanism linking hypertension to both cerebral small vessel disease and Aβ accumulation [1]. Increased PWV may damage the microcirculation due to elevated flow pulsatility [7, 8] and interfere with Aβ clearance by impairing perivascular drainage [9].

Higher PWV has been shown to be associated with poorer cognitive performance (particularly episodic memory and executive functioning) [7, 10] in normally aging samples, mild cognitive impairment (MCI) [11], and cognitive decline over time [12, 13]; however, findings have been inconsistent with some studies reporting the loss of this effect after adjustment for other cardiovascular risk factors [14]. The association between higher PWV and poorer cognition may be particularly salient in carriers of the apolipoprotein E (APOE) ε4 allele [15], the strongest genetic risk factor for late-onset, sporadic AD [16, 17]. APOE ε4 promotes blood-brain barrier (BBB) breakdown [18] and facilitates Aβ accumulation in the brain parenchyma and blood vessel walls [19]. Notably, APOE ε4-mediated neurovascular defects appear to occur prior to neuronal dysfunction and degeneration [18], suggesting that APOE ε4 carriers may be more vulnerable than non-carriers to cerebrovascular dysfunction stemming from adverse vascular changes such as increased PWV [15].

Prior neuroimaging and neuropathological investigations suggest that the co-occurrence of cerebrovascular disease and AD-related changes lowers the threshold at which pathology is expressed as cognitive impairment [20, 21]. These findings suggest that measures of arterial stiffening and in vivo measures of cerebrospinal fluid (CSF) tau and Aβ may interact to reduce cognitive performance in older adults prior to the development of dementia. To test this possibility, we examined the relationships among PWV, genetic and CSF AD risk factors (APOE ε4 genotype, CSF Aβ_42_, and tau), and cognition in a well-characterized sample of older adults free of clinical dementia and stroke. We hypothesized that higher PWV would be associated with poorer episodic memory and executive functioning, especially in individuals at elevated risk of developing AD (i.e., APOE ε4 carriers or CSF tau/Aβ+).

## Materials and methods

### Standard protocol approvals and participant consents

Participants for this cross-sectional study were selected from the ongoing longitudinal study of the University of California, San Diego (UCSD) Shiley-Marcos Alzheimer’s Disease Research Center (ADRC) and ADRC-affiliated research studies. The research protocols were reviewed and approved by the human subject’s review boards at UCSD and/or the VA San Diego Healthcare System. Informed consent was obtained from all participants.

### Participants

Participants were 193 older adults without dementia who had completed clinical, neurological, and neuropsychological evaluations under the ongoing ADRC research study protocol [22, 23]. Non-demented participants are recruited to the ADRC from the community through community outreach events and word-of-mouth and from local practitioners who refer patients with possible memory deficits. The inclusion criteria for the present study included available PWV and neuropsychological test data and no current major physical/medical health problems. The exclusion criteria included a history of major stroke, neurologic disorder, major psychiatric illness, substance abuse, or learning disability. Overall, the participants had a mean age of 74.9 ± 5.9 (mean ± standard deviation) years and a mean of 16.7 ± 2.4 years of formal education, and 53% were women and 10% were Hispanic. Based on the results of the ADRC evaluations, 167 participants were classified as cognitively normal (CN) and 26 as MCI by consensus of a multidisciplinary team that included two senior neurologists and a neuropsychologist. Forty percent of the participants had at least one copy of the APOE ε4 allele. This included 64/167 (38%) CN participants and 14/26 (54%) MCI participants. CSF was available for 123 participants (64.7% of the sample; 103 CN, 20 MCI). The use of anticoagulant medication was a contraindication for lumbar puncture (see Table 1 for participant characteristics across the entire sample; see supplemental tables comparing the characteristics of APOE ε4+ versus APOE ε4− participants (Supplemental Table 1) and tau/Aβ_42_+ versus tau/Aβ_42_− participants (Supplemental Table 2)). Notably, there were no significant group differences between APOE ε4+ and APOE ε4− participants or between tau/Aβ_42_+ and tau/Aβ_42_− participants, in terms of PWV.

**Table 1:** Participant demographics and clinical characteristics

Variable	Total sample, *N* = 193, mean ± SD or number (%)
Age	74.9 ± 5.9
Female	102 (53%)
Education	16.7 ± 2.4
APOE ε4+	78 (40%)
Hispanic	18 (10%)
DRS	138.9 ± 4.3
MMSE	29.1 ± 1.3
CDR sum of boxes	0.3 ± 0.7
GDS	1.2 ± 1.7
Clinical Dx: normal	167 (87%)
Clinical Dx: MCI	26 (13%)
FSRP (%)	10.3 ± 7.9
Systolic BP	131.7 ± 18.6
Diastolic BP	80.4 ± 11.2
Pulse pressure	51.2 ± 13.9
PWV	8.9 ± 2.1
TIA	3 (2%)
Atrial fibrillation	20 (10%)
Diabetes	12 (6%)
CVD	27 (14%)
Smoking	4 (2%)
Antihypertensive medication use	94 (49%)
BMI	26.0 ± 5.6
N with CSF measures	123 (64%)
Interval from LP to PWV collection (years)	1.2 ± 0.9
Aβ_42_	812.2 ± 378.6
Tau	358.8 ± 190.1
AD-like tau/Aβ_42_ ratio	42 (34%)

*Abbreviations*: *CN* cognitively normal, *MCI* mild cognitive impairment, *SD* standard deviation, *APOE* apolipoprotein E, *DRS* Dementia Rating Scale, *MMSE* Mini-Mental State Examination, *CDR* Clinical Dementia Rating Scale, *GDS* Geriatric Depression Scale, *FSRP* Framingham Stroke Risk Profile, *bp* blood pressure. Pulse pressure was calculated as systolic blood pressure minus diastolic blood pressure. *PWV* pulse wave velocity, *CVD* cardiovascular disease, *BMI* body mass index, *CSF* cerebrospinal fluid, *Tau* total tau; *AD* Alzheimer’s disease, *Aβ* amyloid beta

### Neuropsychological evaluation

The comprehensive neuropsychological test battery included measures of global cognitive function (*Dementia Rating Scale*, *Mini Mental State Examination (MMSE)*, *Clinical Dementia Rating (CDR)*), memory (*Wechsler Memory Scale-Revised (WMS-R) Visual Reproduction* and *Logical Memory, California Verbal List Learning Test (CVLT)*), language (*Multilingual Naming Test (MiNT)*, *letter fluency (FAS)*, *category fluency (animals, fruits, vegetables)*, *Pyramids and Palm Trees*), executive functions and attention (*Modified Wisconsin Card Sorting Test*, *Color-Word Interference Test (CWIT)*, *Trail Making Test Parts A and B*, *Wechsler Adult Scale of Intelligence-Revised (WAIS-R) Digit Symbol Substitution Test*, *WMS-R Digit Span*), and visuospatial abilities (*Wechsler Intelligence Scale for Children-Revised (WISC-R) Block Design Test, WMS-R Visual Reproduction copy*).

Cognitive domain scores were generated through principal component analysis (PCA) with varimax rotation of all cognitive test scores (excluding global measures CDR, MMSE, and DRS) using previously described methods [24]. Prior to the PCA, a small percentage of missing scores for each test (< 5% for all tests, except for up to 10% for CWIT and visual reproduction copy) was imputed using a partial means matching approach based on demographics, diagnostic classification, and scores on global cognitive measures and the other cognitive tests. The PCA resulted in five components which were conceptually labeled “memory,” “executive functioning,” “language,” “visuospatial abilities,” and “attention” based on the tests with the highest loadings in each component (Table 2). A cognitive domain score was generated for each component based on the loadings for that component. Test scores from a pool of 146 “robust” normal controls (i.e., diagnosed as normal on their first ADRC evaluation and remained normal for the duration of their participation in the longitudinal study) were submitted to the same component loadings to generate “normal” cognitive domain scores that were used as reference values to “normalize” (i.e., convert cognitive domain scores to z-scores) the scores of the current study participants. The robust normal control participants were selected from the overall ADRC sample in a separate process not associated with the current study; however, 90 of the 146 (62%) robust normal control participants were included in the present study.

**Table 2:** Principal component analysis factor loadings

Measure	Memory	Executive	Language	Attention	Visuospatial
Logical memory—immediate	0.78	0.14	0.35	0.09	0.16
Logical memory—delayed	0.82	0.14	0.30	0.10	0.16
CVLT—trials 1–5	0.85	0.28	0.11	0.14	0.09
CVLT—short delay free	0.88	0.28	0.07	0.10	0.06
CVLT—long delay free	0.88	0.26	0.09	0.08	0.06
Visual reproduction—immediate	0.49	0.35	0.19	0.09	0.59
Visual reproduction—delay	0.64	0.21	0.10	0.07	0.38
Trail A	−0.13	−0.72	−0.27	−0.15	−0.21
Trail B	−0.27	−0.77	−0.14	−0.18	−0.18
Digit symbol	0.32	0.75	0.13	0.20	0.13
CWIT—inhibition	−0.22	−0.76	−0.12	−0.08	−0.05
WCST—categories	0.30	0.48	0.16	−0.01	0.31
Letter fluency	0.25	0.5	0.37	0.42	−0.21
Category fluency	0.41	0.43	0.49	0.34	−0.04
MiNT	0.22	0.25	0.78	0.25	0.07
Pyramids and Palm Trees	0.23	0.25	0.74	−0.03	0.26
Visual reproduction—copy	0.14	0.17	0.09	0.11	0.82
Block design	0.19	0.60	0.36	0.20	0.40
Digit span—forward	0.08	0.09	0.05	0.89	0.08
Digit span—backward	0.16	0.34	0.20	0.70	0.18

*Abbreviations*: *CVLT* California Verbal Learning Test, *CWIT* Color-Word Interference Test, *WCST* Wisconsin Card Sorting Test, *MiNT* Multilingual Naming Test

### Vascular risk assessment: Framingham Stroke Risk Profile (FSRP)

Estimated 10-year stroke risk (as a percentage) was derived using the revised Framingham Stroke Risk Profile (FSRP) [25] which was calculated using exponential equations with sex-specific baseline survival rates and sex-specific beta coefficients for the following risk factors: age, systolic blood pressure, diabetes mellitus, history of cardiovascular disease, atrial fibrillation, cigarette smoking, and use of antihypertensive medications. Systolic blood pressure was the average of three blood pressure readings. Diabetes was defined as self-reported diabetes, use of an anti-diabetic therapy, or casual blood glucose ≥ 200 mg/dL. History of cardiovascular disease (coronary artery disease [myocardial infarction, angina pectoris, coronary insufficiency], intermittent claudication, or cardiac failure) and atrial fibrillation were determined by clinical interview, physical exam, and review of outside medical care records and laboratory studies. Current smoking (yes/no) was based on self-report. The use of antihypertensive medications was ascertained through medication review. Height and weight were recorded, and body mass index (BMI) was calculated with the following formula: weight in kilograms/height in meters squared.

### Pulse wave velocity

Aortic-femoral PWV was measured with the SphygmoCor XCEL device (AtCor Medical, Sydney, NSW, Australia) using well-validated procedures [26, 27] performed by trained operators. All measurements were collected with the participant in the supine position after 10 min of rest. Femoral pulse was measured using a blood pressure cuff around the upper thigh. Carotid pulse was simultaneously measured with a tonometer manually applied at the neck. On average, three PWV measurements were recorded, and the mean value was used in statistical analyses.

### Biomarker acquisition and analysis

Participants received a research lumbar puncture with standardized procedures, preanalytical preparation of CSF, and storage of CSF as previously described [28] and in accordance with the recommended best practices [29]. In brief, CSF (15–25 mL) was collected by routine lumbar puncture early in the morning after overnight fasting. Samples were processed, aliquoted into 500 μL fractions in polypropylene microtubes, snap-frozen, and stored at − 80 °C until assayed. Lumbar punctures were generally conducted within 1 year of the PWV measurements for the vast majority of the participants, although we included CSF data collected within 3 years of the baseline visit given research showing the stability of CSF biomarkers over several years [30].

CSF AD biomarkers examined were beta-amyloid (Aβ42), total tau (Tau), and the ratio of tau over beta-amyloid (tau/Aβ_42_). A small portion of cases (*n* = 18) had biomarkers analyzed in the facilities of ADx (Ghent, Belgium) using ELISA assays developed by ADx and commercialized by EUROIMMUN AG (Lubeck, Germany). The remaining cases’ samples (*n* = 106) were analyzed locally at UCSD using the automated Lumipulse platform using assays developed with established monoclonal antibodies (Fujirebio Inc.) [31]. Weighted Deming regression of a set of 113 CSF samples analyzed on both platforms demonstrated high correlation (Pearson’s R of .87 for Aβ42 and .95 for Tau), so the resulting equations were used to transform ADx values into native Lumipulse space for analyses. A cutpoint for biomarker positivity in Lumipulse space was derived from CSF samples from a larger cohort of 462 unique participants at the UCSD ADRC (ranging from cognitively normal to severely demented). The ratio of tau/Aβ_42_ was used as it appears to provide the best subdivision of individuals without dementia into those with and without pre-clinical AD [32]. A 2-component mixture model was fit to the bimodal distribution of the log-transformed tau/Aβ_42_ ratio using the mixtools [33] package version 1.2.0 for R, and the cutpoint of tau/Aβ_42_ > 0.52 was chosen as the value at which an individual was equally likely to belong to either component of the bimodal distribution [34]. This cutpoint is highly consistent with a published cutpoint (tau/Aβ_42_ > 0.54) for AD biomarker positivity using the Lumipulse assays, derived against clinical read of amyloid PET scans and validated in multiple cohorts [31].

### Statistical analysis

Demographics and clinical characteristics between those with and without CSF were compared by Student’s t-test and chi-squared tests as appropriate. Relationships between PWV and performance in each cognitive domain were analyzed with separate linear models adjusted for age, sex, education, and FSRP. These analyses were then repeated with APOE genotype or CSF AD biomarker status (+/-) added to explore the interaction of PWV and AD risk markers (APOE ε4+; tau/Aβ_42_+) on cognition (while adjusting for age, sex, education, and FSRP).

Additional post hoc analyses were performed to determine whether significant results were retained in models including the individual FSRP components in place of the overall FSRP. That is, in cases where there were significant results, we reran the primary analyses described above but in place of overall FSRP instead adjusted for systolic blood pressure, history of diabetes, cardiovascular disease and/or atrial fibrillation, current smoking, and use of antihypertensive medication. We also reran these analyses with the individual FSRP components listed above while adjusting for two more vascular risk factors: BMI and history of transient ischemic attack (TIA).

## Results

### Associations of PWV with participant characteristics

**Fig. 1:**
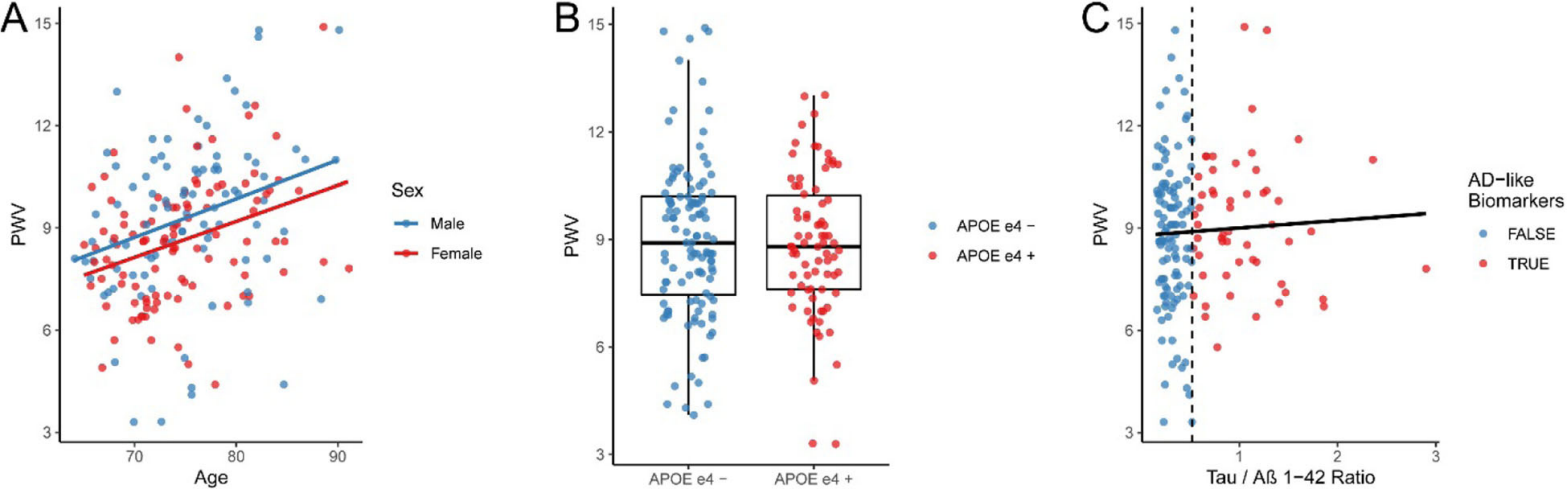
PWV is associated with age and sex but does not differ by APOE ε4 or AD CSF biomarker status. **A** PWV is shown as a function of age for male and female participants. Regression lines fit separately by sex show nearly identical slopes, with men showing consistently higher PWV across the age range. **B** Boxplot of PWV as a function of APOE genotype shows no difference between those with an APOE ε4 allele (ε2ε4, ε3ε4, ε4ε4) and those without (ε2ε2, ε2ε3, ε3ε3). **C** PWV is plotted as a function of AD biomarker level (tau/Aβ ratio). A regression line shows no association. The dashed vertical line represents the biomarker positivity threshold (> 0.52)

Across the cohort, there was a significant increase in PWV with increasing age in both men and women (*p* < 0.001), with no evidence of an interaction between sex and age (*p* = .89). Overall, men had higher PWV than women (*p* < 0.001) (Fig. 1A). There was no significant association between PWV and APOE ε4 status (Fig. 1B), nor between PWV and the level of CSF Tau or Aβ_42_, or the tau/Aβ_42_ ratio (Fig. 1C).

### Associations of PWV with cognitive test performance

**Fig. 2:**
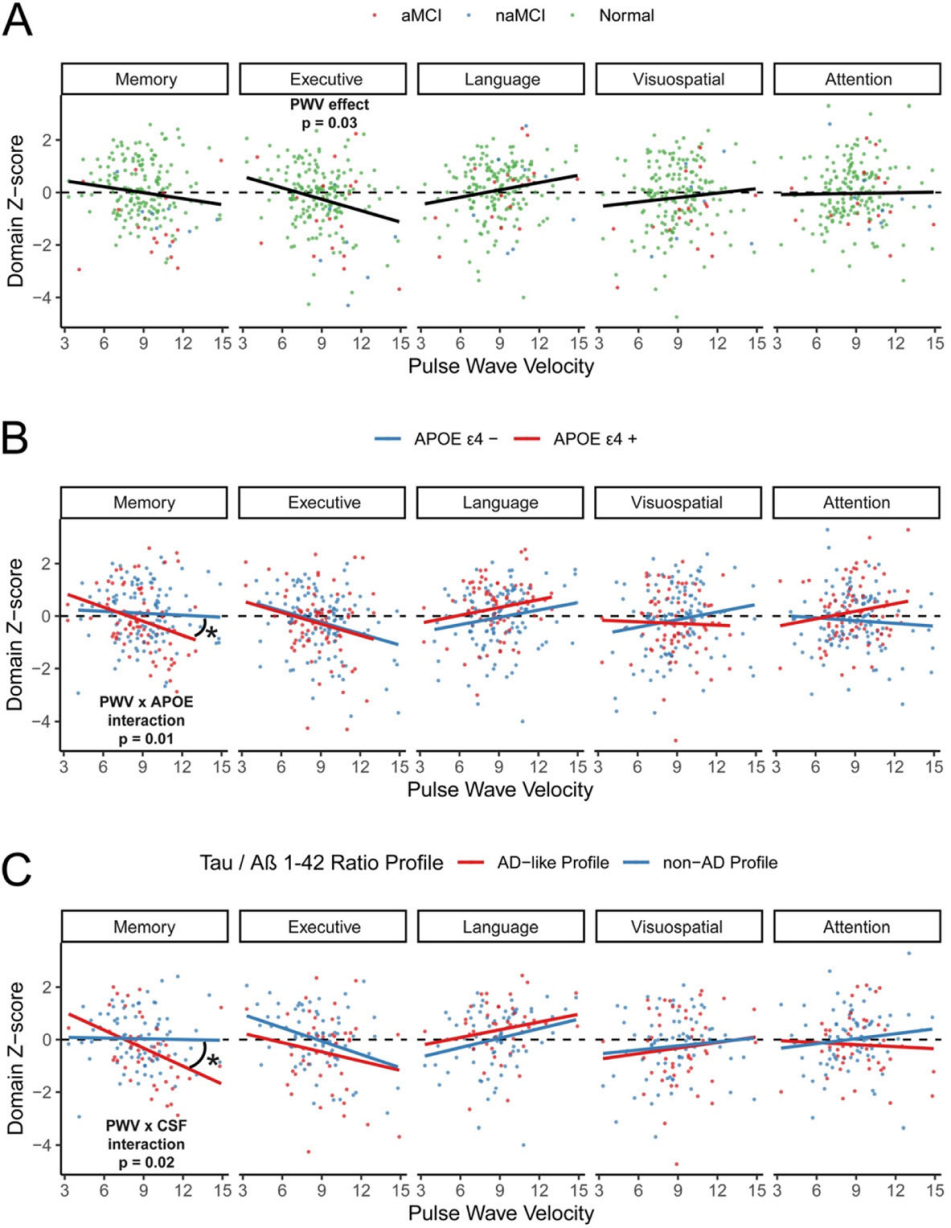
PWV predicts executive function beyond age and sex and interacts with APOE genotype and AD CSF biomarkers to predict memory performance. **A** Cognitive domain z-scores are shown as a function of PWV. Regression models adjusted for age, sex, education, and FSRP (excluding age points) demonstrate a significant main effect of PWV on executive function (*p* = 0.03). **B** The same cases were classified based on APOE genotype as APOE ε4+ (ε2ε4, ε3ε4, ε4ε4) or APOE ε4 − (ε2ε2, ε2ε3, ε3ε3). In regression models examining the interaction of PWV and APOE genotype, a significant interaction (*p* = 0.01) suggested that PWV was more predictive of memory scores in those that were APOE ε4+. **C** In a subset of patients with CSF biomarkers, individuals were classified into AD-like and non-AD profiles based on the tau/Aβ_1–42_ ratio. In regression models examining the interaction of PWV and CSF AD biomarker status, a significant interaction (*p* = 0.02) suggests that PWV was similarly more predictive of memory z-scores in those that were AD-like in their profile

Performance on each of the cognitive domain scores is presented as a function of PWV in Fig. 2A. After adjusting for age, sex, education, and FSRP, PWV was significantly negatively associated with the executive domain score (β = −0.09 ± 0.04, *p* = .03), but not with the memory (β = −0.03 ± 0.04, *p* = .43), language (β = 0.06 ± 0.04, *p* = .07), attention (β = 0.03 ± 0.04, *p* = .42), or visuospatial abilities (β = 0.04 ± 0.05, *p* = .31) domain scores (Fig. 2A).

### Interaction of APOE genotype with PWV on cognitive test performance

Performance on each of the cognitive domain scores is presented as a function of PWV separately for APOE ε4+ and ε4− participants in Fig. 2B. After adjusting for age, sex, education, and FSRP, there was a significant interaction between PWV and APOE genotype on the memory domain score (interaction *p* = .01) reflecting a larger decrease in performance with increasing PWV for the ε4+ participants than for the ε4− participants. There was no interaction between PWV and APOE genotype for the executive functioning (*p* = .73), language (*p* = .86), attention (*p* = .13), or visuospatial abilities (*p* = .21) domain scores (Fig. 2B).

### Interaction of AD biomarkers with PWV on cognitive test performance

Performance on each of the cognitive domain scores is presented as a function of PWV separately for AD biomarker+ and AD biomarker− participants (defined by the tau/Aβ_42_ ratio cutoff) in Fig. 2C. After adjusting for age, sex, education, and FSRP, there was a significant interaction between PWV and AD biomarker positivity on the memory domain score (interaction *p* = .01) reflecting a larger decrease in performance with increasing PWV for the AD biomarker+ participants than for the AD biomarker− participants. There was no interaction between PWV and AD biomarker positivity for the executive functioning (*p* = .69), language (*p* = .97), attention (*p* = .23), or visuospatial abilities (*p* = .83) domain scores (Fig. 2C).

When additional post hoc analyses including individual vascular risk factors in place of overall FSRP score were performed, the results remained similar. That is, the significant main effect of the pulse wave velocity on executive function, and the significant interactions of pulse wave velocity and both APOE ε4 status and CSF AD biomarker status on memory, remained significant (see Supplemental Tables 3, 4, and 5 for detailed results).

## Discussion

We found that increased arterial stiffness measured through carotid-femoral PWV is associated with poorer performance on tests of executive functioning and memory in a well-characterized sample of older adults free of clinical dementia or stroke. The relationship between PWV and executive functioning was independent of the degree of AD risk as determined by APOE genotype or CSF AD biomarkers. In contrast, the relationship between elevated PWV and reduced memory performance was only present in at-risk participants by virtue of the APOE ε4 genotype or an above threshold CSF tau/Aβ ratio. Notably, the observed relationships between PWV and cognition were apparent even after adjusting for important demographic factors and overall cardiovascular risk estimated by the FSRP.

Arterial stiffening increases with advancing age, particularly when cardiovascular disease risk factors are present [35]. This increase in arterial stiffening results in higher PWV and pressure pulsatility, a reduction of the normal protective gradient of increasing stiffness moving from aorta to periphery, transmission of potentially harmful pulsatile energy, and reactive changes to microvascular function and structure that may compromise blood flow [8]. High-flow organs such as the brain may be particularly vulnerable to these secondary changes to the microcirculation and reduced perfusion [8]. Consistent with this possibility, we found that elevated PWV, a reflection of increased arterial stiffness, was associated with poorer executive function regardless of the degree of AD risk. Executive functioning has been repeatedly shown to decline in the context of normal aging [36], and indeed, “normal” cognitive aging has been conceptualized as a selective vulnerability in frontal-subcortical executive processes [37, 38]. Our results suggest that arterial stiffening may contribute to this age-related decline (see [39]). Interestingly, however, we observed a relationship between PWV and executive functioning even after adjusting for age. This suggests that elevated PWV may relate to executive decline over and beyond normal, expected age-related changes [39], and this finding is consistent with prior evidence demonstrating that vascular damage preferentially affects frontally mediated executive abilities [40, 41].

Our finding that elevated PWV interacts with APOE genotype and CSF tau/Aβ_42_ ratio to influence memory function is consistent with previous research suggesting that high pulse pressure and vascular risk factors may interact with genetic risk for AD and markers of AD-related pathology to confer an increased risk of cognitive decline [39, 42, 43]. For example, a previous study found that APOE ε4 carriers demonstrated poorer cognitive performance than non-carriers among participants with higher aortic PWV [15]. The authors proposed that APOE ε4 carriers may be more vulnerable to vascular changes given a loss or reduction of normal protective mechanisms that may be compromised due to existing neurovascular dysfunction and increasing neuropathology. It is possible that there is a threshold effect such that the adverse impact of higher PWV on cognition in at-risk adults may emerge at PWV values that are not detrimental to cognition in older adults with low risk of AD [15].

Our results are also consistent with previous research that has shown reduced regional cerebral blood flow and increased regional cerebrovascular resistance in older adults with elevated vascular risk and in those with MCI and AD [44–46]. These cerebrovascular changes were observed in the subcortical, frontal, and medial temporal regions, and they were linked to poorer cognitive abilities. Impaired cerebrovascular regulation and cerebral hypoperfusion may be most harmful in these brain regions because they are among the most metabolically active, are supplied by terminal arteries, and thereby are susceptible to the effects of small vessel disease, and may be affected by Aβ-mediated vasoconstriction [15, 46]. Our finding of associations between elevated PWV and reduced memory performance in those with AD risk (i.e., APOE e4+ or AD CSF biomarker+) dovetail with this prior work and may reflect the vulnerability of the frontal and hippocampal regions to vascular dysfunction and accumulating ischemic insults [47, 48].

The present results contribute to a growing body of research that suggests vascular vulnerabilities interact with AD pathophysiology to increase the risk of neurodegeneration [39, 43] and cognitive decline [39, 42]. We and others have previously shown that elevated vascular risk burden influences the clinical expression of AD [21] whereby the overlap of cerebrovascular disease and Aβ deposition in the brain lowers the threshold at which an individual with accumulating neuropathology develops cognitive impairment [21, 49]. The two-hit hypothesis of AD proposed by Zlokovic states that vascular disease may contribute to AD pathophysiology through blood-brain barrier breakdown resulting in the leakage of neurotoxic molecules into the tissue and/or through chronic hypoperfusion [2]. It also has been suggested that cerebrovascular abnormalities may impair Aβ clearance via impairment of perivascular drainage [9, 50] and may also directly lead to neurodegeneration independent of cerebral amyloidosis [4]. Indeed, greater arterial stiffening has been associated with the presence of multiple forms of neuropathology including white matter disease, cerebral microbleeds, and tau and Aβ deposition [1, 4]. Given that vascular risk factors such as arterial stiffening are potentially modifiable, their association with cognition suggests that in some cases, cognitive impairment and dementia may be prevented and/or delayed [51]. Targeting arterial stiffening and other vascular risk factors through lifestyle and/or pharmacological interventions may be a promising way to facilitate brain health in aging.

Although PWV has been associated with white matter hyperintensity volume (WMH) [1], studies suggests that WMH only partially mediates the effect of hypertension on cognitive performance [52]. A recent study, for example, showed that microvascular dysfunction measured as a composite of MRI WMH volume and biomarkers of microvascular disease (e.g., plasma soluble intercellular adhesion molecule-1 [sICAM-1] and soluble vascular adhesion molecule-1 [sVCAM-1]) explained only 16.2% of the effect of aortic stiffness on cognitive function [53]. Studies examining WMH volume and other vascular risk factors such as diabetes also have not found an imaging metric that fully explains the association between the risk factor and cognitive performance [54, 55]. Taken together, these studies highlight the importance of examining vascular risk factors in and of themselves rather than only the downstream end-organ damage they may cause [55].

The strengths of this study include the use of a well-validated carotid-femoral PWV measurement to assess arterial stiffening, administration of a comprehensive neuropsychological test battery that allowed empirical derivation of cognitive domain scores, and examination of CSF tau and Aβ biomarkers to detect AD pathology in non-demented older adults. In addition, we adjusted for important demographic and vascular risk factor variables that influence cognition including age, sex, and aggregate vascular risk (as well as the individual FSRP components, BMI, and history of TIA in post hoc analyses), and our findings remained statistically significant suggesting PWV is an independent contributor to cognitive performance.

## Limitations

Our study is limited in generalizability given the cohort is a relatively homogeneous sample of older adults who were predominantly White and well-educated with relatively good vascular health. Indeed, the prevalence of FSRP components of cardiovascular disease (14%) and diabetes (6%) in our sample were lower than in the general population over age 65 (24 to 37% for cardiovascular disease and 22 to 23% for diabetes) [56]. It is likely that associations between PWV and cognitive abilities would have been stronger in a sample with a greater vascular risk burden, and these associations should be explored in people with more prominent risk factors in future studies. Nonetheless, our findings suggest that even relatively mild vascular dysfunction may contribute to poorer cognitive function in individuals with elevated AD risk. In addition, the percentage of APOE ε4+ cognitively normal participants in our cohort (38%) is higher than reported in some previously published studies. AD case-control studies combining data from several research groups reported that among Caucasian individuals, approximately 14% of cognitively normal controls were APOE ε4 carriers [57, 58] (although allele frequencies are known to vary based on factors such as sex, ethnicity, and geography [59, 60]). However, the percentage of APOE ε4+ cognitively normal participants in our cohort is very similar to those reported in other AD cohort studies (e.g., Uniform Data Set of the Alzheimer’s Disease Centers [UDS] program and Australian Imaging, Biomarkers and Lifestyle Flagship Study of Ageing [AIBL]) [61]. Notably, there is bias in recruitment for our study and other AD cohort studies that results in enrichment for APOE ε4 carriers, and this limits the generalizability of our findings to the general population [61]. We also do not have available FLAIR WMH data for our cohort, which will be critical to include in future studies to better characterize the presence and extent of vascular disease in our participants. Future longitudinal studies are necessary to determine if APOE genotype and AD biomarkers influence the trajectories of PWV and its relationship to cognitive decline as well as to explore some of the potential mechanisms underlying these relationships.

## Conclusions

The findings of the current study showed that elevated PWV is associated with poorer cognitive performance in older adults, particularly on memory tasks, in those who are at risk for AD. These findings suggest that PWV, a non-invasive method to assess arterial stiffness, should be explored as a potential marker of risk for cognitive decline in older adults. PWV appears to have added predictive value beyond the FSRP and individual FSRP variables, including blood pressure. While previous studies suggest that PWV correlates strongly with fluid-attenuated inversion recovery (FLAIR) MRI WMH measures [1], PWV is much easier and less expensive to acquire than MRI and can be measured and tracked in a clinician’s primary care office. In addition, there are very few contraindications of PWV assessment, whereas many older adults have contraindications to MRI (e.g., pacemakers, metal implants, claustrophobia). Our results also provide a rationale for attempts to improve central vascular function and decrease cerebral pulsatility as an intervention to reduce dementia risk by preventing or slowing the accumulation of AD and vascular neuropathology across the aging spectrum [1, 39].

## Supplementary Information

**Additional file 1: Table S1.** Participant Demographics and Clinical Characteristics by APOE ε4 Status. **Table S2.** Participant Demographics and Clinical Characteristics by AD CSF Biomarker Status. **Table S3.** Post-hoc Exploration of Influence of Individual Vascular Risk Variables in Place of Overall Framingham Stroke Risk Profile: Results of Models Examining Main Effect of PWV on Executive Function. **Table S4.** Post-hoc Exploration of Influence of Individual Vascular Risk Variables in Place of Overall Framingham Stroke Risk Profile: Results of Models Examining Interaction of PWV and APOE ε4 Status on Memory. **Table S5.** Post-hoc Exploration of Influence of Individual Vascular Risk Variables in Place of Overall Framingham Stroke Risk Profile: Results of Models Examining Interaction of PWV and CSF AD Biomarker Status on Memory.

## Data Availability

Anonymized datasets analyzed in the current study are available to qualified investigators on reasonable request.

## Abbreviations

AD: Alzheimer’s disease
APOE: Apolipoprotein E
CSF: Cerebrospinal fluid
Aβ: Beta-amyloid
PWV: Pulse wave velocity
MCI: Mild cognitive impairment
BBB: Blood-brain barrier
UCSD: University of California, San Diego
ADRC: Alzheimer’s Disease Research Center
CN: Cognitively normal
MMSE: Mini-Mental State Examination
CDR: Clinical Dementia Rating
WMS-R: Wechsler Memory Scale-Revised
CVLT: California Verbal List Learning Test
MiNT: Multilingual Naming Test
CWIT: Color-Word Interference Test
WAIS-R: Wechsler Adult Scale of Intelligence-Revised
WISC-R: Wechsler Intelligence Scale for Children-Revised
PCA: Principal component analysis
FSRP: Framingham Stroke Risk Profile
BMI: Body mass index
TIA: Transient ischemic attack
