# Supplementary material for: Arterial stiffening acts synergistically with APOE genotype and AD biomarker status to influence memory in older adults without dementia
Source: Alzheimers Res Ther. 2021 Jul 1;13:121. doi: 10.1186/s13195-021-00851-2 (PMC8246656; doi:10.1186/s13195-021-00851-2)
Supplement: Supplementary file 1 — Additional file 1: Table S1. Participant Demographics and Clinical Characteristics by APOE ε4 Status. Table S2. Participant Demographics and Clinical Characteristics by AD CSF Biomarker Status. Table S3. Post-hoc Exploration of Influence of Individual Vascular Risk Variables in Place of Overall Framingham Stroke Risk Profile: Results of Models Examining Main Effect of PWV on Executive Function. Table S4. Post-hoc Exploration of Influence of Individual Vascular Risk Variables in Place of Overall Framingham Stroke Risk Profile: Results of Models Examining Interaction of PWV and APOE ε4 Status on Memory. Table S5. Post-hoc Exploration of Influence of Individual Vascular Risk Variables in Place of Overall Framingham Stroke Risk Profile: Results of Models Examining Interaction of PWV and CSF AD Biomarker Status on Memory. [file 13195_2021_851_MOESM1_ESM.docx]

**Supplemental Table 1: Participant Demographics and Clinical Characteristics by APOE ε4 Status**

| Variable | APOE ε4-  N=115 | APOE ε4+  N=78 | P value |
| --- | --- | --- | --- |
|  | Mean ± SD  or number (%) | Mean ± SD  or number (%) |  |
| Age | 75.7 ± 5.9 | 73.7 ± 5.7 | **.02** |
| Female | 61 (53%) | 41 (53%) | .95 |
| Education | 16.7 ± 2.4 | 16.7 ± 2.4 | .99 |
| APOE ε4+ | 0 (0%) | 78 (100%) | --- |
| Hispanic | 14 (13%) | 4 (5%) | .12 |
| DRS | 139.1 ± 3.9 | 138.6 ± 4.9 | .44 |
| MMSE | 29.1 ± 1.3 | 29.0 ± 1.4 | .60 |
| CDR sum of boxes | 0.2 ± 0.5 | 0.4 ± 0.9 | **.04** |
| GDS | 1.2 ± 1.7 | 1.2 ± 1.7 | .77 |
| Clinical Dx: Normal | 103 (90%) | 64 (82%) | .14 |
| Clinical Dx: MCI | 12 (10%) | 14 (18%) | .14 |
| FSRP (%) | 10.7 ± 8.2 | 9.6 ± 7.4 | .34 |
| Systolic BP | 133.7 ± 18.8 | 128.7 ± 17.9 | .07 |
| Diastolic BP | 80.6 ± 11.9 | 80.2 ± 10.2 | .83 |
| Pulse Pressure | 53.0 ± 14.4 | 48.5 ± 12.9 | **.03** |
| PWV | 9.0 ± 2.2 | 8.9 ± 2.0 | .68 |
| TIA | 1 (1%) | 2 (3%) | .37 |
| Atrial Fibrillation | 12 (10%) | 8 (10%) | .97 |
| Diabetes | 9 (8%) | 3 (4%) | .27 |
| CVD | 16 (14%) | 11 (14%) | .97 |
| Smoking | 2 (2%) | 2 (3%) | .70 |
| Antihypertensive Medication Use | 55 (48%) | 39 (50%) | .77 |
| BMI | 26.3 ± 6.5 | 25.6 ± 3.9 | .43 |
| N with CSF measures | 67 (58%) | 56 (72%) | .06 |
| Interval from LP to PWV collection (yrs) | 1.3 ± 0.9 | 1.2 ± 0.8 | .51 |
| Aβ_42_ | 885.0 ± 357.9 | 725.2 ± 387.4 | **.02** |
| Tau | 341.4 ± 217.6 | 379.7 ± 150.0 | .27 |
| AD-like Tau/Aβ_42_ Ratio | 13 (19%) | 29 (52%) | **< .001** |

CN = Cognitively normal; MCI = mild cognitive impairment; SD = standard deviation; APOE = apolipoprotein E; DRS = Dementia Rating Scale; MMSE = Mini Mental State Examination; CDR = Clinical Dementia Rating scale; GDS = Geriatric Depression Scale; FSRP = Framingham Stroke Risk Profile; bp = blood pressure; PWV = pulse wave velocity; CVD = cardiovascular disease; BMI = body mass index; CSF = cerebrospinal fluid; tau = total tau; AD = Alzheimer’s disease; Aβ = amyloid beta

**Supplemental Table 2: Participant Demographics and Clinical Characteristics by AD CSF Biomarker Status**

| Variable | Non-AD Biomarker Profile  N=81 | AD-like Biomarker Profile  N=42 | P value |
| --- | --- | --- | --- |
|  | Mean ± SD  or number (%) | Mean ± SD  or number (%) |  |
| Age | 73.1 ± 4.7 | 75.1 ± 5.9 | .051 |
| Female | 39 (48%) | 23 (55%) | .48 |
| Education | 17.0 ± 2.5 | 16.5 ± 2.4 | .24 |
| APOE ε4+ | 27 (33%) | 29 (69%) | **< .001** |
| Hispanic | 7 (9%) | 2 (5%) | .44 |
| DRS | 139.6 ± 3.0 | 136.8 ± 5.3 | **< .001** |
| MMSE | 29.0 ± 1.3 | 28.9 ± 1.4 | .48 |
| CDR sum of boxes | 0.1 ± 0.4 | 0.6 ± 1.2 | **.002** |
| GDS | 1.1 ± 1.4 | 1.1 ± 1.4 | .85 |
| Clinical Dx: Normal | 75 (93%) | 28 (67%) | **< .001** |
| Clinical Dx: MCI | 6 (7%) | 14 (33%) | **< .001** |
| FSRP (%) | 8.4 ± 4.5 | 11.3 ± 9.3 | **.03** |
| Systolic BP | 131.6 ± 19.6 | 131.2 ± 20.5 | .93 |
| Diastolic BP | 81.1 ± 12.6 | 80.3 ± 10.5 | .75 |
| Pulse Pressure | 50.3 ± 14.7 | 50.9 ± 15.3 | .84 |
| PWV | 8.6 ± 2.4 | 9.3 ± 2.0 | .13 |
| TIA | 1 (1%) | 0 (0%) | .99 |
| Atrial Fibrillation | 5 (6%) | 3 (7%) | .84 |
| Diabetes | 8 (10%) | 1 (2%) | .16 |
| CVD | 10 (12%) | 5 (12%) | .94 |
| Smoking | 2 (2%) | 2 (5%) | .50 |
| Antihypertensive Medication Use | 39 (48%) | 19 (45%) | .78 |
| BMI | 26.9 ± 7.1 | 25.2 ± 4.1 | .15 |
| N with CSF measures | 81 (100%) | 42 (100%) | --- |
| Interval from LP to PWV collection (yrs) | 1.3 ± 0.8 | 1.1 ± 0.9 | .38 |
| Aβ_42_ | 957.7 ± 380.1 | 531.7 ± 149.1 | **< .001** |
| Tau | 275.3 ± 100.5 | 519.9 ± 217.8 | **< .001** |
| AD-like Tau/Aβ_42_ Ratio | 0 (0%) | 42 (100%) | **^---^** |

CN = Cognitively normal; MCI = mild cognitive impairment; SD = standard deviation; APOE = apolipoprotein E; DRS = Dementia Rating Scale; MMSE = Mini Mental State Examination; CDR = Clinical Dementia Rating scale; GDS = Geriatric Depression Scale; FSRP = Framingham Stroke Risk Profile; bp = blood pressure; PWV = pulse wave velocity; CVD = cardiovascular disease; BMI = body mass index; CSF = cerebrospinal fluid; tau = total tau; AD = Alzheimer’s disease; Aβ = amyloid beta

**Supplemental Table 3: Post-hoc Exploration of Influence of Individual Vascular Risk Variables in Place of Overall Framingham Stroke Risk Profile: Results of Models Examining Main Effect of PWV on Executive Function**

|  | Model 1: Primary model adjusted for FSRP | | Model 2: Adjusted for FSRP components | | Model 3: Adjusted for FSRP components, TIA, and BMI | |
| --- | --- | --- | --- | --- | --- | --- |
| Variable | Estimate +/- Std. Error | P Value | Estimate +/- Std. Error | P Value | Estimate +/- Std. Error | P Value |
| **PWV** | -0.09 ± 0.04 | **.03** | -0.09 ± 0.04 | **.04** | -0.10 ± 0.04 | **.04** |
| Age | -0.05 ± 0.02 | **.002** | -0.07 ± 0.01 | **< .001** | -0.07 ± 0.02 | **< .001** |
| Sex | -0.36 ± 0.17 | **.03** | -0.42 ± 0.17 | **.02** | -0.45 ± 0.18 | **.01** |
| Education | 0.02 ± 0.03 | .60 | 0.02 ± 0.03 | .60 | 0.02 ± 0.04 | .62 |
| FSRP | -2.25 ± 1.29 | .08 |  |  |  |  |
| Systolic BP (per 10 mmHg) |  |  | -0.01 ± 0.05 | .83 | -0.03 ± 0.05 | .51 |
| Diabetes |  |  | -0.25 ± 0.36 | .49 | -0.22 ± 0.38 | .56 |
| CVD |  |  | 0.16 ± 0.25 | .53 | 0.15 ± 0.26 | .56 |
| Afib |  |  | -0.51 ± 0.28 | .07 | -0.40 ± 0.30 | .19 |
| Smoking |  |  | -0.75 ± 0.58 | .20 | -0.72 ± 0.58 | .21 |
| Anti-HTN Medication |  |  | -0.16 ± 0.17 | .35 | -0.19 ± 0.18 | .28 |
| TIA |  |  |  |  | -0.59 ± 0.70 | .39 |
| BMI |  |  |  |  | 0.02 ± 0.01 | .22 |

PWV = pulse wave velocity; FSRP = Framingham Stroke Risk Profile; BMI = body mass index; TIA = transient ischemic attack; BP = blood pressure; mmHG = millimeters per mercury; CVD = cardiovascular disease; Afib = atrial fibrillation; HTN = hypertension

Bold font indicates p-values < 0.05.

**Supplemental Table 4: Post-hoc Exploration of Influence of Individual Vascular Risk Variables in Place of Overall Framingham Stroke Risk Profile: Results of Models Examining Interaction of PWV and APOE ε4 Status on Memory**

|  | Model 1: Primary model adjusted for FSRP | | Model 2: Adjusted for FSRP components | | Model 3: Adjusted for FSRP components, TIA, and BMI | |
| --- | --- | --- | --- | --- | --- | --- |
| Variable | Estimate +/- Std. Error | P Value | Estimate +/- Std. Error | P Value | Estimate +/- Std. Error | P Value |
| PWV | 0.04 ± 0.05 | .41 | 0.04 ± 0.05 | .47 | 0.06 ± 0.05 | .25 |
| APOE ε4+ | 1.34 ± 0.68 | **.0495** | 1.40 ± 0.69 | **.04** | 1.53 ± 0.70 | **.03** |
| **PWV x APOE ε4+ interaction** | -0.19 ± 0.07 | **.01** | -0.19 ± 0.08 | **.01** | -0.21 ± 0.08 | **.006** |
| Age | -0.01 ± 0.02 | .35 | -0.02 ± 0.01 | .18 | -0.02 ± 0.01 | .12 |
| Sex | 0.86 ± 0.15 | **< .001** | 0.87 ± 0.16 | **< .001** | 0.96 ± 0.17 | **< .001** |
| Education | 0.04 ± 0.03 | .24 | 0.04 ± 0.03 | .24 | 0.04 ± 0.03 | .25 |
| FSRP | -0.68 ± 1.20 | .57 |  |  |  |  |
| Systolic BP (per 10 mmHg) |  |  | 0.01 ± 0.04 | .84 | -0.01 ± 0.05 | .77 |
| Diabetes |  |  | 0.01 ± 0.34 | .96 | 0.15 ± 0.36 | .68 |
| CVD |  |  | 0.03 ± 0.23 | .90 | -0.04 ± 0.24 | .88 |
| Afib |  |  | 0.06 ± 0.26 | .81 | -0.11 ± 0.28 | .70 |
| Smoking |  |  | 0.34 ± 0.54 | .53 | 0.44 ± 0.54 | .41 |
| Anti-HTN Medication |  |  | -0.17 ± 0.16 | .31 | -0.15 ± 0.16 | .37 |
| TIA |  |  |  |  | 0.91 ± 0.65 | .16 |
| BMI |  |  |  |  | -0.003 ± 0.01 | .82 |

PWV = pulse wave velocity; FSRP = Framingham Stroke Risk Profile; BMI = body mass index; TIA = transient ischemic attack; BP = blood pressure; mmHG = millimeters per mercury; CVD = cardiovascular disease; Afib = atrial fibrillation; HTN = hypertension

Bold font indicates p-values < 0.05.

**Supplemental Table 5: Post-hoc Exploration of Influence of Individual Vascular Risk Variables in Place of Overall Framingham Stroke Risk Profile: Results of Models Examining Interaction of PWV and CSF AD Biomarker Status on Memory**

|  | Model 1: Primary model adjusted for FSRP | | Model 2: Adjusted for FSRP components | | Model 3: Adjusted for FSRP components, TIA, and BMI | |
| --- | --- | --- | --- | --- | --- | --- |
| Variable | Estimate +/- Std. Error | P Value | Estimate +/- Std. Error | P Value | Estimate +/- Std. Error | P Value |
| PWV | 0.02 ± 0.05 | .67 | 0.001 ± 0.06 | .98 | -0.002 ± 0.06 | .98 |
| AD Biomarker+ | 1.27 ± 0.81 | .12 | 1.53 ± 0.82 | .07 | 0.60 ± 0.91 | .08 |
| **PWV x AD Biomarker+ Interaction** | -0.19 ± 0.07 | **.02** | -0.20 ± 0.09 | **.02** | -0.21 ± 0.10 | **.03** |
| Age | -0.007 ± 0.02 | .74 | -0.01 ± 0.02 | .58 | -0.01 ± 0.02 | .59 |
| Sex | 0.84 ± 0.19 | **< .001** | 0.77 ± 0.20 | **< .001** | 0.76 ± 0.21 | **< .001** |
| Education | 0.06 ± 0.04 | .13 | 0.06 ± 0.04 | .10 | 0.07 ± 0.04 | .10 |
| FSRP | -1.18 ± 1.79 | .51 |  |  |  |  |
| Systolic BP (per 10 mmHg) |  |  | 0.06 ± 0.05 | .21 | 0.07 ± 0.06 | .19 |
| Diabetes |  |  | 0.23 ± 0.39 | .56 | 0.24 ± 0.39 | .55 |
| CVD |  |  | -0.16 ± 0.31 | .61 | -0.16 ± 0.31 | .61 |
| Afib |  |  | -0.29 ± 0.40 | .48 | -0.28 ± 0.41 | .49 |
| Smoking |  |  | 0.43 ± 0.53 | .42 | 0.40 ± 0.54 | .46 |
| Anti-HTN Medication |  |  | -0.24 ± 0.20 | .23 | -0.24 ± 0.21 | .25 |
| TIA |  |  |  |  | -0.29 ± 1.21 | .81 |
| BMI |  |  |  |  | -0.006 ± 0.02 | .68 |

PWV = pulse wave velocity; CSF = cerebrospinal fluid; AD = Alzheimer’s diseae; FSRP = Framingham Stroke Risk Profile; BMI = body mass index; TIA = transient ischemic attack; BP = blood pressure; mmHG = millimeters per mercury; CVD = cardiovascular disease; Afib = atrial fibrillation; HTN = hypertension

Bold font indicates p-values < 0.05.
